# Semantics in Support of Biodiversity Knowledge Discovery: An Introduction to the Biological Collections Ontology and Related Ontologies

**DOI:** 10.1371/journal.pone.0089606

**Published:** 2014-03-03

**Authors:** Ramona L. Walls, John Deck, Robert Guralnick, Steve Baskauf, Reed Beaman, Stanley Blum, Shawn Bowers, Pier Luigi Buttigieg, Neil Davies, Dag Endresen, Maria Alejandra Gandolfo, Robert Hanner, Alyssa Janning, Leonard Krishtalka, Andréa Matsunaga, Peter Midford, Norman Morrison, Éamonn Ó. Tuama, Mark Schildhauer, Barry Smith, Brian J. Stucky, Andrea Thomer, John Wieczorek, Jamie Whitacre, John Wooley

**Affiliations:** 1 The iPlant Collaborative, University of Arizona, Tucson, Arizona, United States of America; 2 University of California, Berkeley, Berkeley, California, United States of America; 3 Department of Ecology and Evolutionary Biology and the CU Museum of Natural History, University of Colorado at Boulder, Boulder, Colorado, United States of America; 4 Department of Biological Sciences, Vanderbilt University, Nashville, Tennessee, United States of America; 5 University of Florida, Florida Museum of Natural History, Gainesville, Florida, United States of America; 6 Research Informatics, California Academy of Sciences, San Francisco, California, United States of America; 7 Gonzaga University, Computer Science, Spokane, Washington, United States of America; 8 Alfred Wegener Institute, Helmholtz Centre for Polar and Marine Research, Bremerhaven, Germany; 9 University of California, Berkeley, Gump South Pacific Research Station, Moorea, French Polynesia; 10 GBIF Norway, Natural History Museum, University in Oslo, Oslo, Norway; 11 LH Bailey Hortorium, Department of Plant Biology, Cornell University, Ithaca, New York, United States of America; 12 Biodiversity Institute of Ontario, University of Guelph, Guelph, ON, Canada; 13 School of Information Resources and Library Science, University of Arizona, Tucson, Arizona, United States of America; 14 Biodiversity Institute and Ecology & Evolutionary Biology, The University of Kansas, Lawrence, Kansas, United States of America; 15 University of Florida, Gainesville, Florida, United States of America; 16 Ecology and Evolutionary Biology, University of Kansas, Lawrence, Kansas, United States of America; 17 The BioVeL Project, School of Computer Science, The University of Manchester, Manchester, United Kingdom; 18 GBIF Secretariat, Copenhagen, Denmark; 19 National Center for Ecological Analysis and Synthesis, Santa Barbara, California, United States of America; 20 Department of Philosophy, University at Buffalo, Buffalo, New York, United States of America; 21 Department of Ecology and Evolutionary Biology, University of Colorado, Boulder, Colorado, United States of America; 22 Graduate School of Library and Information Science, University of Illinois at Urbana-Champaign, Urbana-Champaign, Illinois, United States of America; 23 3101 VLSB, Museum of Vertebrate Zoology, University of California, Berkeley, Berkeley, California, United States of America; 24 Informatics Branch, Information Technology Office, National Museum of Natural History, Smithsonian Institution, Washington, DC, United States of America; 25 University of California San Diego, La Jolla, California, United States of America; King Abdullah University of Science and Technology, Saudi Arabia

## Abstract

The study of biodiversity spans many disciplines and includes data pertaining to species distributions and abundances, genetic sequences, trait measurements, and ecological niches, complemented by information on collection and measurement protocols. A review of the current landscape of metadata standards and ontologies in biodiversity science suggests that existing standards such as the Darwin Core terminology are inadequate for describing biodiversity data in a semantically meaningful and computationally useful way. Existing ontologies, such as the Gene Ontology and others in the Open Biological and Biomedical Ontologies (OBO) Foundry library, provide a semantic structure but lack many of the necessary terms to describe biodiversity data in all its dimensions. In this paper, we describe the motivation for and ongoing development of a new Biological Collections Ontology, the Environment Ontology, and the Population and Community Ontology. These ontologies share the aim of improving data aggregation and integration across the biodiversity domain and can be used to describe physical samples and sampling processes (for example, collection, extraction, and preservation techniques), as well as biodiversity observations that involve no physical sampling. Together they encompass studies of: 1) individual organisms, including voucher specimens from ecological studies and museum specimens, 2) bulk or environmental samples (e.g., gut contents, soil, water) that include DNA, other molecules, and potentially many organisms, especially microbes, and 3) survey-based ecological observations. We discuss how these ontologies can be applied to biodiversity use cases that span genetic, organismal, and ecosystem levels of organization. We argue that if adopted as a standard and rigorously applied and enriched by the biodiversity community, these ontologies would significantly reduce barriers to data discovery, integration, and exchange among biodiversity resources and researchers.

## Introduction

The loss of biodiversity is a major societal issue of our time, ultimately impacting the need for food, fuel, fiber, and animal feed [1]–[3]. Recognition of the accelerating loss of biodiversity has prompted immediate, global action, including initiatives such as the Convention on Biological Diversity (CBD) – an agreement between 150 countries dedicated to sustainable development [4] – and the Intergovernmental Platform on Biodiversity and Ecosystem Services (IPBES). These initiatives require scientific research into underlying biological, physical, and chemical processes to develop predictive models and inform policy decisions. Trustworthy data about past and present biodiversity are essential to achieve these goals [5], and the Group on Earth Observation Biodiversity Observation Network (GEO-BON) was established as the international organization to coordinate these efforts [6].

Assembling the data sets needed for global biodiversity initiatives remains challenging. Biodiversity data are highly heterogeneous, including information about organisms, their morphology and genetics, life history and habitats, and geographical ranges. These data almost always either contain or are linked to spatial, temporal, and environmental data. Biodiversity science seeks to understand the origin, maintenance, and function of this variation and thus requires integrated data on the spatiotemporal dynamics of organisms, populations, and species, together with information on their ecological and environmental context. Biodiversity knowledge is generated across multiple disciplines, each with its own community practices. As a consequence, biodiversity data are stored in a fragmented network of resource silos, in formats that impede integration. The means to properly describe and interrelate these different data sources and types is essential if such resources are to fulfill their potential for flexible use and re-use in a wide variety of monitoring, scientific, and policy-oriented applications [5].

Even the most basic quantification of biodiversity, such as accurately accounting for the species on the planet or representing the geographic distribution of those species, remains frustratingly incomplete [7], [8]. New approaches, such as high-throughput DNA sequencing of environmental samples, promise to accelerate a quantitative assessment of biodiversity [9], including the vast and still largely unexplored diversity found among microbes. However, these approaches also create new challenges, because they may bypass traditional description, naming, and classification processes, leading to a disconnect between names and sequences [10]. Nevertheless, advances in molecular biology and the ‘big data’ they generate are stimulating the adoption of new information technologies that erode the separation among data, interpretation, and publishing through new dissemination methods that support linked data and rich media [11]–[13].

All of these advances underscore the urgent need for improved approaches to describe the many ways that biodiversity scientists capture and assemble data as well as the semantics of the data. Resilient standards and ontologies will be central in addressing this need and will help scientists make use of heterogeneous data in a reliable, harmonized manner – one that relies wherever possible on automatic reasoning rather than on *ad hoc* manual comparison and assembly of data. The use of ontologies has become widespread in fields such as biomedicine, where they enhance data discovery and access, data interoperability, and knowledge discovery (e.g., [14]–[16]). The adoption of similar tools by the biodiversity science community would allow the use of big data approaches [17] to build a dynamic picture of population and community assemblages across space and time and to test hypotheses of how organisms function and interact within a given niche, ecosystem, or region.

In this paper we report on the ongoing development of ontologies that describe sampling and observing processes of 1) organisms, including ecological voucher specimens and museum specimens that underpin taxonomic knowledge, 2) bulk and environmental samples that contain DNA, other molecules, and often multiple organisms, particularly microbes, and 3) survey-based ecological observations that often do not include the archiving of physical samples. Although not exhaustive, these three examples span much of the breadth of biodiversity sampling and observing processes. Existing ontologies and standards (described in more detail in the following sections) were not designed to describe and integrate data across these processes, and the need to do so motivated the creation of the Biological Collections Ontology (BCO), a semantic resource representing the central notions of sampling, specimen collection, and observations. Herein, we present and describe the BCO, including its relationships to other biological ontologies – in particular the Environment Ontology (ENVO), a common framework for describing environmental information [18], [19], and the Population and Community Ontology (PCO), which models collections of biological entities and their interactions. Finally, we discuss how this set of ontologies can be applied to real-life biodiversity use cases and argue for their adoption by the biodiversity community. These ontologies, particularly the BCO and PCO, are currently under development, and our goal is to provide them to the scientific community in an early but still usable form, in order to promote continued collaborative development.

Throughout this paper, we distinguish between ontologies and vocabularies. The former model a knowledge domain, defining the classes of entities, their properties, and the relations between them, whereas the latter are typically flat collections of terms with definitions but with little semantics. Ontology terms (classes and relations/predicates) herein are printed in italics, prefixed by the corresponding acronym (e.g., BCO:*material sample*). We report only on terms with a BCO, ENVO, or PCO prefix. Although some authors of this paper were involved in the development of many other terminologies described herein, we do not report on the development of those terminologies. Terms from the Darwin Core (DwC) vocabulary are not italicized, because they do not come from an ontology. However, they are prefaced with the namespace abbreviation “dwc:” which is shorthand for http://rs.tdwg.org/dwc/terms/.

### The diversity of biodiversity data – the need for integration

Because biodiversity science spans many disciplines and ranges in scale from molecules to ecosystems, biodiversity data come in many forms. Initial development of the BCO is focused on ways to facilitate integration of data from museum specimen collections, bulk and environmental samples that contain many molecules and organisms, and survey-based ecological observations that do not retain a physical sample.

**Museum and herbarium specimens** are the primary physical evidence that document biodiversity via collected and preserved organisms or their parts. These specimens are the subject of the morphological observations, descriptions, and publications that have underpinned biological taxonomy for over 250 years [20]. We recognize that evolution, phylogenetic systematics, and taxonomy play a fundamental role in organizing biological information, and for at least the past two decades, researchers have been trying to clarify the distinct logical models underlying various biological classifications [21]–[27]. Classification yields concepts and taxa that represent scientific hypotheses; placing those models into an ontology is beyond the scope of this paper, but a discussion of the applicability of ontologies to taxonomy can be found in [28].

New entities – for example, digital images or tissue subsamples – can be derived from museum specimens. This derivation may involve procedures that are destructive, as in the case of tissue harvesting for DNA extraction. In many cases, subsamples find their way into other types of collections, such as cryo-facilities, that are often housed and databased independently from the source collection. Comprehensive biodiversity surveys, such as the Moorea Biocode Project (described in more detail in the Discussion), along with many smaller-scale projects, can be enhanced by the ability to track objects and data across multiple resources and communicate relationships derived from specimen subsampling and distribution to multiple physical or digital repositories. Such tracking is not easily accommodated by current data infrastructure and is one driving use case for the development of the BCO.

**Figure 1:**
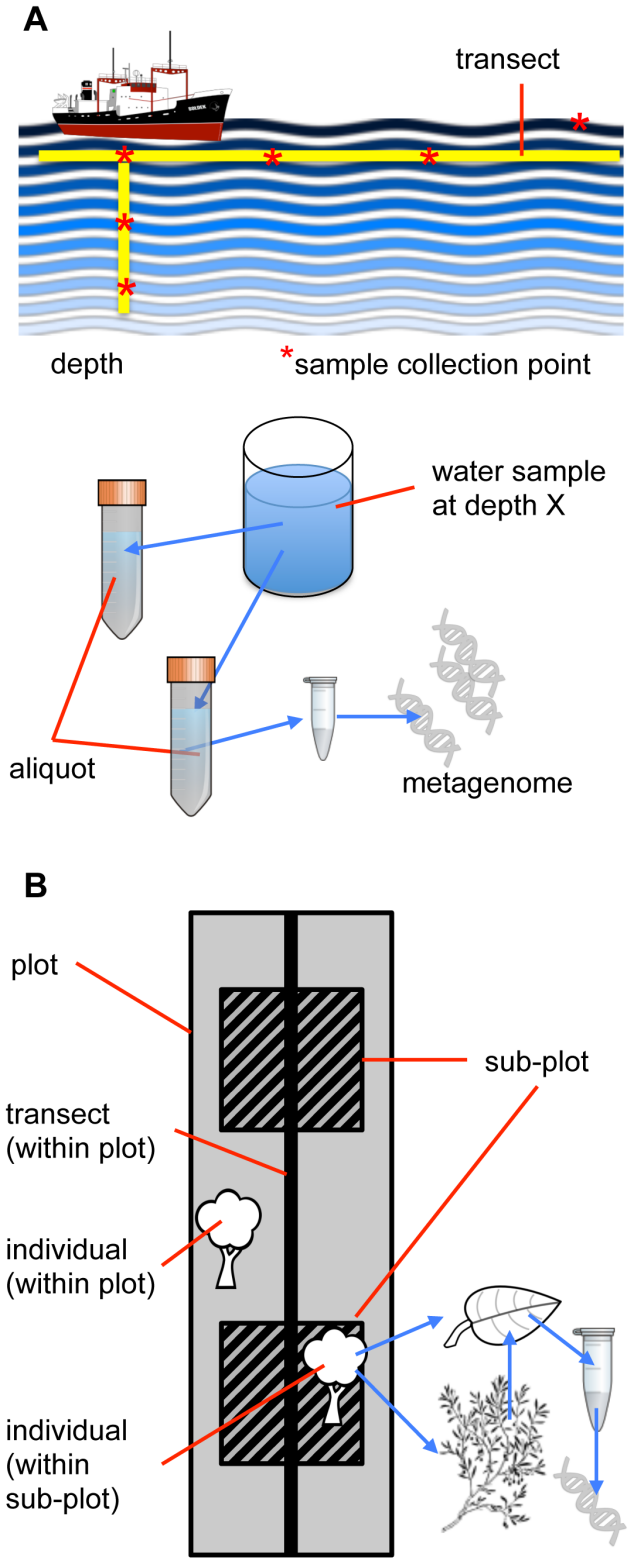
Structured sampling schemes. (**A**) Biological sampling can be structured in both space and time. Environmental sampling of ocean water often includes sampling along a transect, with samples collected at multiple depths at each location. Additionally, each sample of water collected may be subsampled for metagenomic analysis or measuring chemical content. (**B**) Sampling schemes in ecological studies are often nested and may include plot; subplot or transect within plot; individual within plot, subplot, or transect; organ (e.g., leaf) within individual; tissue within organ; and DNA or mineral (e.g., C or N) within tissue. DNA extracted from a leaf of a tree that is present in a sub-plot may therefore be characterized by environmental features of the plot.

**Environmental samples** are of growing importance for high throughput analyses based on advances in DNA sequencing. The field of metagenomics, for example, employs molecular techniques to address the genetic and taxonomic composition of whole communities of organisms (e.g., those present in the gut of an organism or in a sample of soil or water), as well as the function of those communities [29]. We use the term ‘environmental sampling’ as it is commonly used, although bulk sampling is perhaps a more accurate term. The key point for our purposes here is that the samples are known to contain many different organisms or parts thereof, often including DNA from multicellular organisms plus entire microbial organisms, not whether the samples are from an abiotic material (e.g., soil or water samples) or from the microbiome of an organism (e.g., gut content of a fish or mesophyll tissue of a leaf). In reality, this condition applies to museum specimens too, but, in contrast to traditional museum specimen workflows, environmental sampling explicitly seeks to characterize the mixed communities within the sample. In the case of microbes, these samples often have a species composition that is poorly characterized in terms of traditional taxonomy, as many microbes cannot be cultured. For microbial studies, the environmental context, such as the temperature, pressure, and other physicochemical properties of the original material sampled, is particularly important. The sequence data derived from an environmental sample should inherit the data describing the location, host taxon (in the case of a microbiome), or environmental conditions of the sample. Tracking metadata associated with environmental samples is further complicated by structured sampling protocols, such as ocean sampling shown in Figure 1A. The need to semantically describe the biological and environmental components of metagenomic samples provides a driving use case for the PCO and ENVO, while the need to link data across sampling events – for example, a metagenomic sampling of an animal's gut and the museum specimen of that animal – has motivated the development of the BCO. Environmental sampling is a key component of the Genomic Observatories Network [8], [30] use case, described in more detail in the Discussion.

**Ecological surveys** provide a third, distinct source of biodiversity data. Survey methods are heterogeneous, but they are often based on a defined time spent quantifying the distribution and abundance of species or individuals within a particular spatial range, rather than single point occurrences. In contrast to museum collections, many ecological studies are based on observations or measurements taken from samples that are neither collected nor archived. Like environmental samples, survey targets may exhibit nested relationships with other features, such as a leaf coming from a plant located in a subplot within a plot (Figure 1B). As a result of this spatial nesting, environmental variables associated with a plot may also be associated with a leaf collected within that plot and with the DNA extracted from that leaf. This type of nested observation or sampling requires the same sort of metadata tracking through a chain of events that was described above for museum and environmental sampling. Database implementations, such as TRY [31] or BIEN [32], use relational databases to successfully model the complexity of ecological sampling, but interpretation of the tables and their attributes is limited to the internal schema of these databases. Expressing ecological data as linked data in Resource Description Format (RDF), using terms drawn from ontologies such as ENVO, PCO, and BCO, can provide the semantic framework needed for automated access to, and reasoning over, what is potentially a huge source of networked data.

### Community Development Processes and Current State of Biodiversity Standards

Although there is still no widely accepted terminology or standard that spans all aspects of biodiversity sampling and observing, there is a long history of community-developed vocabularies and standards for particular aspects of biodiversity data, particularly for museum collection information. We highlight some of those efforts with an eye to how their specific limitations inspired the development of the BCO and ongoing efforts in the PCO and ENVO.

The Biodiversity Information Standards (TDWG) organization is a community dedicated to the development of standards for the exchange of biological/biodiversity data. TDWG has ratified and maintains the Darwin Core (DwC) [33] and Access to Biological Collections Data (ABCD) [34] standards. DwC is a relatively small (∼200) set of terms and definitions – in the spirit of minimum information standards – that was explicitly developed with no class-property hierarchical structure. This was due to both considerations of simplicity and a lack of mature standards for expressing semantics at the time. ABCD includes terms in common with, and mapped to, DwC, but it has a hierarchical structure and many more terms (∼1200). Although it aims to define the semantics of all of its terms, it is not specified as an ontology and lacks a subject-predicate-object format. Both DwC and ABCD have extensions to increase the scope of data they can cover (e.g., DNA collections) and both have been described formally as XML schemas. DwC has been formally described in RDF (dwc-rdf) thus facilitating re-use of terms, but ABCD currently is not available in RDF. While DwC and ABCD represent an important advance in the standardization of biodiversity data, neither is designed to provide the kind of semantics or knowledge modeling needed for robust logical inference. At an even more basic level, many term definitions in the DwC vocabulary have broad definitions that can be interpreted in multiple ways, seriously limiting the ability to use these terms in automated reasoning (e.g., dwc∶Taxon is defined as “the category of information pertaining to taxonomic names, taxon name usages, or taxon concepts” and the DwC type term dwctype∶Taxon is defined as “a resource describing an instance of the Taxon class”).

At their 2006 annual conference, TDWG initiated an ontology effort to build a semantic framework tied to Life Sciences Identifiers (LSIDs) [35]. The first draft of this ontology, named the TDWG LSID Ontology (http://rs.tdwg.org/ontology/), was intended to guide the further development of standards for biodiversity information. For a variety of reasons, the development of the TDWG LSID ontology later stalled and was discontinued. A new ontology with a more limited focus on the Darwin Core terminology was presented at the TDWG conference in 2011. This product was dubbed Darwin Core Semantic Web (DSW) [36]. DSW provides pairs of inverse object properties that can be used to relate instances of DwC-defined classes. It also codifies a particular outlook on the relationships among the DwC classes that includes differentiating between an individual organism, the presence of an organism at a location (the DSW definition of dwc∶Occurrence), and the evidence that documents that presence, such as specimens [37]. DSW provides a semantic framework for reasoning over biodiversity data, but is limited to the context of the DwC terminology and is thus not sufficiently general to cover many of the use cases driving the development of the BCO.

TDWG efforts have primarily focused on the description of objects in museum collections, with some attention to observational data, whereas the Genomic Standards Consortium (GSC) [38] has focused on the annotation of genetic sequence data, including those obtained from environmental samples. The GSC's standards are specified in the Minimum Information about any (x) Sequence (MIxS) [39] family of metadata checklists. MIxS consists of checklists for genome/metagenome sequences (MIGS/MIMS) and genetic marker sequences (MIMARKS), with shared descriptors across all three checklists, checklist-specific descriptors, and a suite of environment-specific descriptor “packages”. These lists provide an avenue for contextualizing sequences at the time of collection or submission to repositories and, where possible, specify the use of terms from community-sanctioned ontologies such as ENVO. MIGS and MIMS are formalized in an XML schema (the Genomic Contextual Data Markup Language or GCDML) [40] but currently are not available as RDF vocabularies. One limitation of the MIxS standards is that the metadata do not contain a sufficient semantic framework for relating genomic and metagenomic samples to individual organisms, identification instances (e.g., species names), and the sampling processes from which they were derived. BCO seeks to address this gap.

Parallel to the efforts described above, a task group established in 2010 by the Global Biodiversity Information Facility (GBIF) to explore options for the implementation of Knowledge Organization Systems for biodiversity information standards [41], [42] proposed to initiate a closer integration between the TDWG standards and the Open Biological and Biomedical Ontologies (OBO) Foundry framework, specifically by proposing to adopt some of the OBO Foundry principles [43]. Based on that proposal, in 2011, the NSF-funded Research Coordination Network for the Genomic Standards Consortium (RCN4GSC) [44], [45] began a series of meetings to reconcile discrepancies between terms in the DwC and the MIxS standards [46]. This activity was meant to help harmonize vocabularies used to describe museum collections data and metagenomic biodiversity assays. The vocabulary alignment meetings recognized inconsistencies in the use of fundamental terms such as ‘sample’, ‘specimen’, and ‘occurrence’. In response, the RCN4GSC organized a Semantics of Biodiversity (SoB) workshop in Lawrence, Kansas in May of 2012 [47]. SoB brought together a range of domain experts to comment on a proposal for aligning terms within a larger framework, using the Basic Formal Ontology (BFO) [48], [49] and OBO Foundry principles as a guide.

Building on the SoB event, the Biocode Commons Ontology Hackathon, supported by the RCN4GSC and the BiSciCol project, was held at GSC14 in Oxford [47] to formalize the concepts outlined at the SoB workshop as an ontology. Initial investigations revealed that existing OBO Foundry ontologies, such as the Gene Ontology (GO) [50], [51], Sequence Ontology (SO) [52], [53], or Ontology for Biomedical Investigations (OBI) [54] – while providing some classes relevant to the biodiversity domain – do not model concepts like museum specimens or environmental sampling and their relationships to the entities derived from them. As a result, a decision was made to develop the BCO further as a separate ontology.

At both the SoB and the Biocode Commons workshops, participants were aware that ontology development would need to support existing standards such as DwC and MIxS. However, they chose to model biological sampling and processes *de novo*, in order to avoid specific shortcomings of the existing standards, such as the lumping of collected specimens and observations (i.e. measurements or sightings recorded without a collected specimen) in dwc∶Occurrence. Workshop participants also recognized the need to connect BCO concepts to allied ontologies such as ENVO and PCO and supported continued development within the OBO Foundry framework, including the use of the BFO as an upper level ontology.

Following the GSC14 meeting, the “Biocode Commons” was established as a formal GSC Project to provide the informatics stack for the Genomic Observatories Network [9], [30] – a collaboration of GSC and GEO-BON. The BCO draws heavily on use cases from the Genomic Observatories Network and is working to establish BCO as a key objective of the Biocode Commons. Furthermore, the NSF-funded EAGER: Interoperative Informatics Infrastructure for Biodiversity Research (I3BR; hosted at UCSD with John Wooley as PI) is building on the efforts of the RCN4GSC, TDWG, GSC, and GBIF to support the increased interoperability of molecular and biodiversity standards and syntax and thus enhance semantic interoperability of their data holdings. The BCO represents an important part of this effort, by providing the necessary semantics to model biodiversity data. Going forward, I3BR support will help create task groups to establish the infrastructure for managing ontologies.

## Methods and Results: Ontology Development

### The Biological Collections Ontology (BCO)

This manuscript describes the October 1, 2013 BCO release, which is available to view or download in the Web Ontology Language (OWL) [55] at http://purl.obolibrary.org/obo/bco/releases/2013-10-01/bco.owl (Table 1). The most current stable version of the BCO is always available at http://purl.obolibrary.org/obo/bco.owl and can be browsed via BioPortal at http://bioportal.bioontology.org/ontologies/BCO. The most current production version of the BCO is available at http://bco.googlecode.com/git/src/ontology/bco.owl.

**Table 1:** Metrics on current versions of the BCO, ENVO, and PCO.

Ontology	# of terms: total/in namespace/imported	# of relations: total/subclassOf^1^	# of deprecated terms
Biological Collections Ontology (BCO)	102/42/60^2^	39/24	15
Environment Ontology (ENVO)	1556/1335/221^3^	2077/1868	19
Population and Community Ontology (PCO)	1345/24/1321^4^	20/18	0

1 . For BCO and PCO, the number of relations includes only relations that point to a BCO or PCO term, to adjust for the large proportion of imported terms.

2 . 39 imported from Basic Formal Ontology, 13 imported from Information Artifact Ontology, 10 imported from Ontology for Biomedical Investigations, 1 imported from Common Anatomy Reference Ontology.

3 . 172 imported from Chemical Entities of Biological Interest, 49 from Phenotypic Quality Ontology.

4 . 39 imported from Basic Formal Ontology, 1269 imported from Gene Ontology, 11 imported from Information Artifact Ontology, 2 imported from Common Anatomy Reference Ontology.

Curation of the BCO follows a community development model as practiced by many other OBO Foundry ontologies. Initial development was described above in “*Community Development Processes and Current State of Biodiversity Standards*,” and subsequent development is being hosted in a public repository at http://code.google.com/p/bco/. Anyone is welcome to suggest additions or modifications to the ontology via the Google Code issue tracker, or to join the BCO mailing list (https://groups.google.com/forum/?fromgroups#!forum/bco-discuss). Coordination with other OBO Foundry ontologies takes place via the OBO-discuss mailing list.

**Figure 2:**
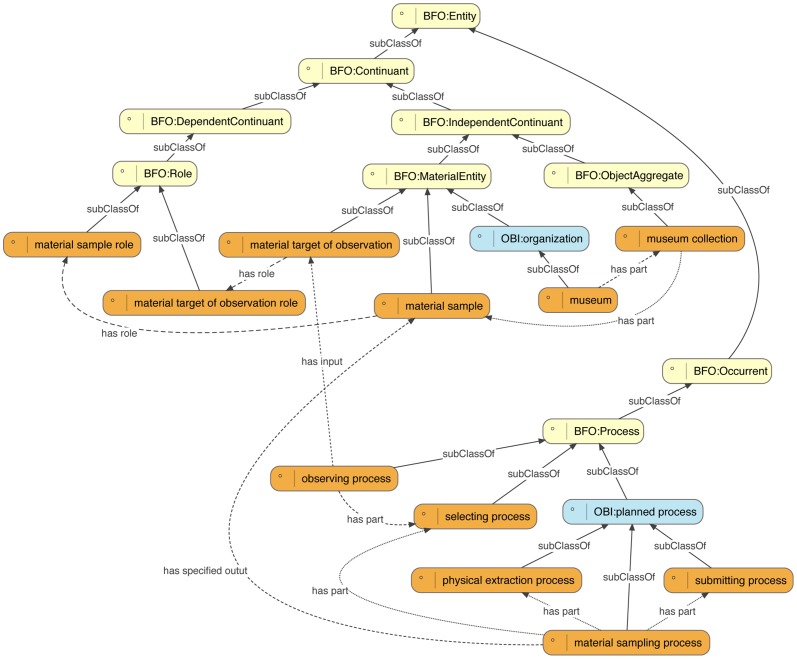
Core terms of the Biological Collections Ontology (BCO) and their relations to upper ontologies. Core BCO terms (in orange) are subclasses of terms from the Basic Formal Ontology (BFO – in yellow) or the Ontology for Biomedical Investigations (OBI – in blue). For example, BCO:*material sample* is a subclass of BFO:*material entity* and has role BFO:*material sample role* (which is a BFO:*role*), while BFO:*material sampling process* is a subclass of OBI:*planned process*, and has as specified output BCO:*material sample*.

**Figure 3:**
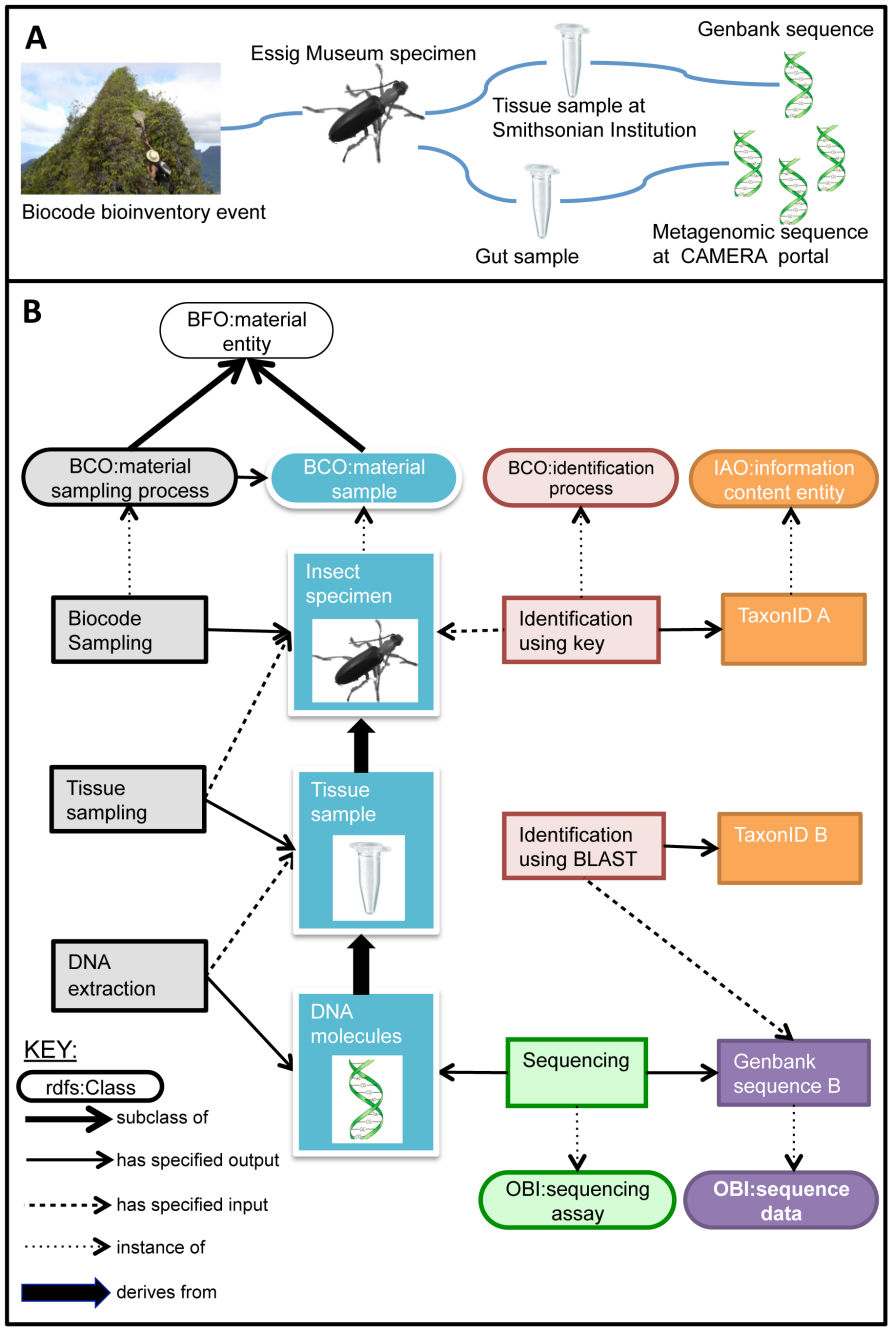
Linking samples and derivatives from the Moorea Biocode project. (**A**) Biodiversity data from the Moorea Biocode project were collected at many different levels that are connected to one another in biologically meaningful ways, such as an Essig Museum specimen collected as part of a Biocode bioinventory event, a tissue sample submitted to the Smithsonian Institution, a metagenomic gut sample collected from the specimen and registered with the CAMERA portal, or DNA extracted from either the tissue or metagenomic sample. (**B**) A graphical representation of how part of the workflow shown in **A** (from field collection to tissue sampling to DNA extraction) can be annotated with terms from multiple, coordinated ontologies and queried via an ontology-based data store. Ontology classes are shown as ovals and instances are shown as rectangles, with instances color-coded to match their parent classes. This figure shows how, for example, TaxonID B resulting from the BLAST identification process on Genbank sequence B can be linked back to the original Moorea Biocode sampling process, or how a chain of inputs and outputs can be used to infer that an instance of DNA molecules is derived from an instance of an insect specimen.

**Figure 4:**
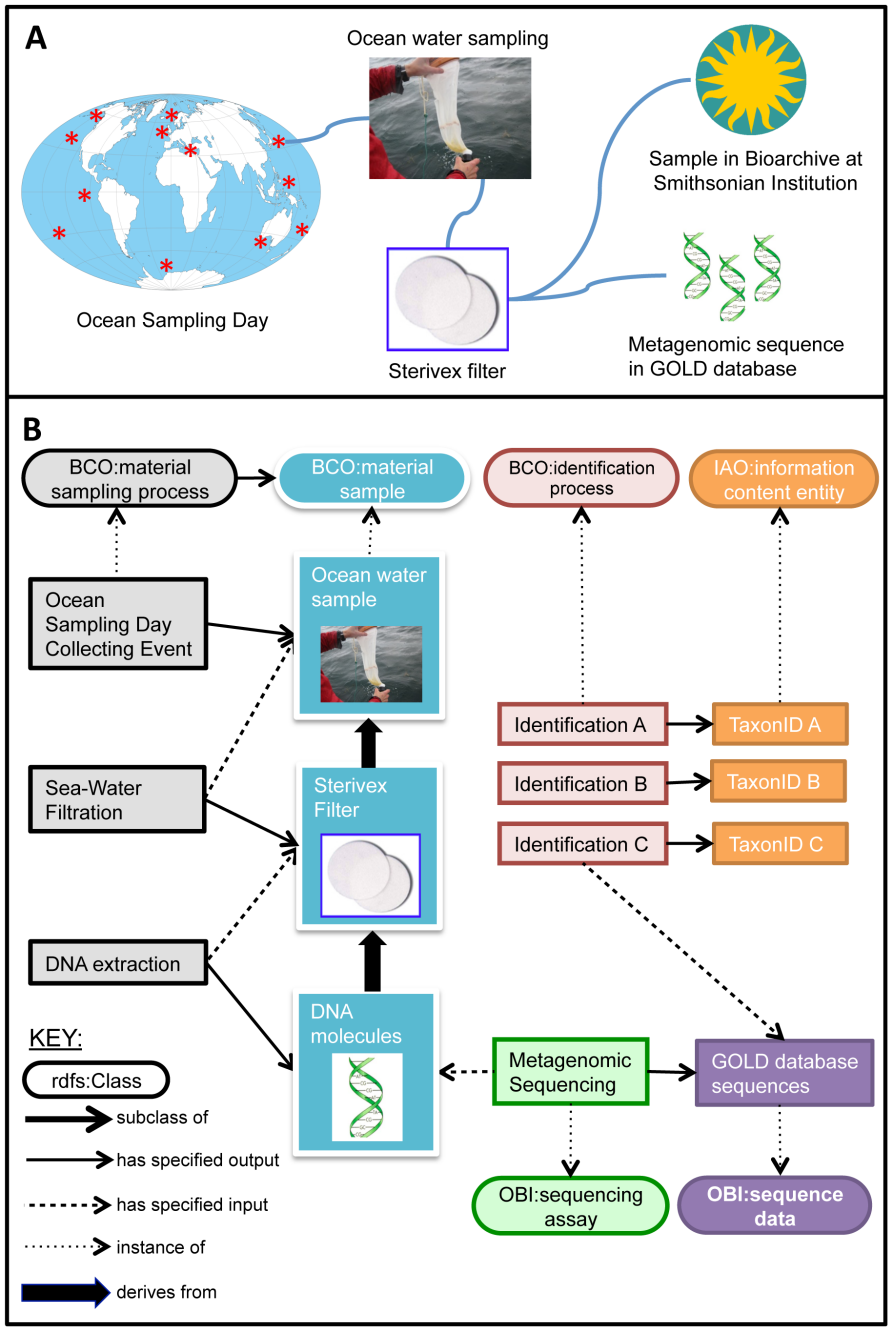
Linking data across sites in the Genomic Observatories network's Ocean Sampling Day. (**A**) Ocean Sampling Day involves the simultaneous sampling of the world's oceans on a single day, as represented by the red stars on the map of the earth. Multiple ocean water sampling processes take place at each location. Those water samples are filtered to produce samples of organismal communities that are submitted to the bioarchive at the Smithsonian Institution. A subsample of the filtered material is analyzed to produce a metagenomic sequence, which may be stored in the Genomes Online Database (GOLD). To be useful in comparative studies, data from each process at each location must be accessible and interpretable. (**B**) A graphical representation of how part of the workflow shown in **A** (from ocean water sampling to filtering to metagenomic sequencing) can be annotated with terms from multiple, coordinated ontologies and queried via an ontology-based data store. Ontology classes are shown as ovals and instances are shown as rectangles, with instances color-coded to match their parent classes. This figure shows how a metagenomic sequence and the taxa associated with it can be linked back to the original Ocean Sampling Day collecting event through a chain of inputs and outputs.

Development in the BCO to date has focused on the terms BCO:*material sample* and BCO:*material sampling process* and related classes (Figure 2). A BCO:*material sample* (Figure 2) is defined as a BFO:*material entity* that is the output of a BCO:*material sampling process* and which has a BCO:*material sample role*. Examples of BFO:*material entities* that may be classified as BCO:*material samples* include a preserved animal in a museum collection (Figure 3A) a portion of ocean water in a jar (Figure 4A), a herbarium specimen, or a fossil specimen. A jar of ocean water takes on or realizes the BCO:*material sample role* by virtue of taking part in a BCO:*material sampling process*. That is, it is selected for study, physically extracted from the environment, and submitted for preservation or study. Because any BFO:*material entity* can realize a BCO:*material sample role* by being the output of some BCO:*material sampling process*, it is the specification of the role that allows entities to be classified as BCO:*material samples*.

If a BCO:*material sampling process* is further carried out on a BCO:*material sample*, the resulting BCO:*material sample* is known colloquially as a subsample. For example, in an experimental process where DNA was extracted from a sample of a microbial community, which was extracted by filtration from a jar of marine water, BCO:*material samples* derived from the jar of marine water can be called subsamples. As the conceptualization of a sample and subsample are very similar (both are BFO:*material entities* that are the product of a BCO:*material sampling process*), we use an instance-level representation of the targets and products of a BCO:*material sampling process* as a means to identify procedural subsamples, without the creation of an explicit subsample class.

BCO:*material sampling process* (Figure 2) is a subclass of OBI:*planned process*, which comes from the Ontology for Biomedical Investigations (OBI) [54]. Three other types of processes are used to define a BCO:*material sampling process*: a BCO:*selecting process* (a planned process by which a person or machine decides that a particular material entity is worthy of collection), a BCO:*physical extraction process* (a planned process that involves removing a material sample from one site to another), and a BCO:*submitting process* (a planned process whereby a person submits a material sample to an organization). A BCO:*material sampling process* is distinguished from a BCO:*observing process* in that a BCO:*observing process* has as output an IAO:*information content entity* (from the Information Artifact Ontology or IAO), rather than a BCO:*material sample*, although both processes have a BCO:*selecting process* as a part. Other processes involved in biodiversity investigations, such as photographing organisms or specimens, will be covered by future versions the BCO, and terminology for modeling species inventories is currently under development.

### The Environment Ontology (ENVO)

This manuscript briefly describes the March 1, 2013 ENVO release, which is available to view or download in OBO format at http://envo.googlecode.com/svn/releases/2013-03-01/envo.obo (Table 1). Herein we focus on the aspects of ENVO that are applicable to biodiversity science, but a more complete description of ENVO, including its curatorial process is available at [19]. The latest version of ENVO can be browsed on the ENVO website (http://www.environmentontology.org/Browse-EnvO). The ontology is versioned in a Google code repository (http://code.google.com/p/envo/) and requests for new classes handled by an associated issue tracker.

ENVO [18] was initiated in 2007 and has been adopted by the GSC. ENVO is a community-developed ontology for the standardized description of the environmental context of any entity of interest. Any instances of a BFO:*material entity*, including instances of PCO:*species*, PCO:*population*, or PCO:*community*, as well as instances of a BFO:*process* may be annotated using ENVO classes. While ENVO classes make no reference to specific locations or to generic geospatial properties, they are naturally linked to geospatial information. Such information may be expressed via resources such as ENVO's sister-project, Gaz, a first step towards an open source gazetteer constructed on ontological principles.

ENVO includes three hierarchies comprising the subclasses of ENVO:*biome*, ENVO:*environmental feature*, and ENVO:*environmental material*, described more fully in [19]. Ideally, when annotating entities with ENVO, classes from each of these hierarchies should be combined to describe an environment from these three different perspectives. An example of a minimal annotation of a pelagic shark observed feeding near a shallow coral reef would include three classes: ENVO:*neritic epipelagic zone biome* (biome), ENVO:*coral reef* (environmental feature), and ENVO:*coastal water* (environmental material).

A future release of ENVO will include classes defining the concepts of habitat and niche, with reference to the relevant concepts in PCO. Following community review, these classes, together with ENVO:*biome*, ENVO:*environmental feature*, and ENVO:*environmental material*, aim to lay a foundation for more refined and standardized handling of these key ecological concepts. Finally, in an effort to enhance their clarity and conform to OBO Foundry principles, ENVO top-level classes are currently being aligned with BFO, and work to establish formal definitions is in progress.

### The Population and Community Ontology (PCO)

This manuscript describes the October 3, 2013 PCO release, which is available to view or download in the Web Ontology Language (OWL) [55] at http://purl.obolibrary.org/obo/pco/releases/2013-10-03/pco.owl (Table 1). The most current stable version of the PCO is always available at http://purl.obolibrary.org/obo/pco.owl and can be browsed via BioPortal at http://bioportal.bioontology.org/ontologies/PCO. The most current production version of PCO is available at http://popcomm-ontology.googlecode.com/svn/trunk/src/ontology/pco.owl.

Curation of the PCO follows the same community development model as described above for the BCO. The requests for new terms and modifications can be made at the issue tracker (http://code.google.com/p/popcomm-ontology/issues/list) or to the PCO mailing list (popcomm-ontology@googlegroups.com).

Development of the PCO presents some special challenges for ontology coordination because, until recently, ontological terminology for populations and communities has been developed in an *ad hoc* manner spread over multiple ontologies. A goal of the PCO project is to coordinate that development by defining terminology for populations and communities in collaboration with the appropriate domain experts and continuing discussions with the curators of other ontologies such as GO, Infectious Disease Ontology (IDO) [56], Phenotypic Quality Ontology (PATO), and NeuroBehavior Ontology [57] about how PCO terminology should be integrated with those ontologies.

The PCO aims to serve the bioinformatics needs of population-based studies such as ecology, evolutionary biology, community healthcare, and clinical biomedical research. Within the context of biodiversity studies, PCO terminology is important for describing multi-organism (e.g., metagenomic or ecological) samples and sampling, as well as for the construction of logical definitions of terms such as niche or habitat (see section on ENVO above).

## Discussion

Many applications of biodiversity science require the collection, integration, and analysis of data from a variety of sources as well as a way to link information about biological entities and their derivatives as materials and data move through various processes and institutions (Table S1). Ontologies offer an opportunity to link data semantically within and across biodiversity sub-disciplines, by creating a unified knowledge model that spans many data types. The BCO – in conjunction with other ontologies such as PCO, ENVO, or OBI – helps to break down the barriers among data silos, enhancing the value of biodiversity data by allowing researchers to query across data sets. We illustrate the complexity of the problem domain and the utility of ontologies by focusing on two specific use cases drawn from the Moorea Biocode Project and the Genomic Observatories Network, as mentioned earlier, but also discuss additional examples from other contexts.

### Tracking samples in a large bio-inventory project: the Moorea Biocode Project

The Moorea Biocode Project aimed to create the first comprehensive inventory of all non-microbial life in a tropical ecosystem by constructing a library of genetic markers (DNA barcodes [58]) and physical identifiers for every species of plant, animal, and fungus on the Pacific island of Moorea [59]. Each step in the Moorea Biocode Project, such as those shown in Figure 3A, follows protocols, has inputs and outputs, and is accompanied by metadata collection. Starting at any step in the chain, researchers need to find and access data/metadata associated with any other step. Figure 3B shows selected ontology terms that can be used to annotate data from the Moorea Biocode Project. For the sake of clarity, Figure 3 does not show every relationship that could or should be annotated in this workflow.

One outcome of the annotation process is to enable a linked data approach [60] by representing relationships among instances and between instances and ontology term identifiers, using uniform resource identifiers (URIs) as globally unique identifiers. The BiSciCol project is implementing such an approach by storing relationships harvested from community-accessible data sets and enabling queries using relevant ontologies. Some examples of the types of queries that could be performed in the context of the Moorea Biocode Project use case include:

1. Show cases where the taxonomic identification determined through morphological keying (e.g., TaxonID A in Figure 3B) differs from that determined through DNA sequencing (e.g., TaxonID B in Figure 3B).
2. List the ENVO:*feature* and other environmental parameters recorded during a Moorea Biocode *sampling process* that are associated with Genbank sequence B.
3. Return all the taxa that have been collected as part of the Moorea Biocode Project and where to find the specimens, DNA samples, and sequences associated with those taxa.

### Coordinating multi-site environmental sampling: Genomic Observatories Network

The Genomics Observatories Network aims to build a global network of research sites, each of which collect and integrate genomic, environmental, and socio-ecological data – all well contextualized by the time and place of collection [9], [30]. Genomic Observatories may be terrestrial, freshwater, or marine, and should support intensive environmental and ecological data collection as part of a long-term commitment to research in that ecosystem. Data from the study sites are digitized for export to global data repositories such as GBIF and the International Nucleotide Sequence Database Collaboration (INSDC).

Ocean Sampling Day (OSD) is an initial project of the Genomic Observatories Network that involves a simultaneous sampling campaign of the world's oceans on the summer solstice of 2014 (Figure 4). The broader EU FP7 Project MicroB3 is developing metadata collection protocols and workflows for OSD [61]. Samples will be characterized for their planktonic and microbial composition as well as their water quality (e.g., optical qualities, dissolved minerals). OSD aims to have standardized metadata that will describe the sampling process, post-capture processing of samples, data generation and analysis, and information on the sampling sites. Because OSD is a global project, it relies heavily on a distributed network of sampling stations (i.e. Genomic Observatories) spanning many countries and institutions, some in extreme environments. Consequently, the ability to relate samples and sampling processes – from the field and the lab – to analyses and publications is a major challenge for project management. The Genomic Observatories Network established the Biocode Commons as an open, collaborative community for building the necessary informatics stack for biodiversity genomics research, such as that of OSD.

Figure 4B describes the inputs and outputs from OSD, including instances of: BCO:*material sample* to track physical samples such as sea water vials, filter discs, and DNA molecules; BCO:*material sampling process* to track events related to these samples; and BCO:*identification process* to track events that lead to taxon name assignments. Examples of the types of queries that could be performed in the context of the OSD use case include:

1. For a given taxon identification, show process metadata related to the relevant ocean-water filtration and collecting event.
2. Return a map of all locations where a given taxon was found on Ocean Sampling Day.
3. Show a list of identified taxa that are found in a given range of environmental conditions.
4. Discover metadata related to the DNA extraction process for a given sequence.

Genetic analysis requires the expense of physical sampling, so consolidating efforts across Genomic Observatories helps to maximize the knowledge gained from these field collections by focusing efforts at scientifically important sites [9]. Realizing the full potential of such an approach, however, requires the linking of data through ontologies, not only within projects like OSD but also between projects and across different scientific fields. Efforts to annotate biodiversity data sets with ontology terms are underway and will be available through the BCO code repository in the future.

### Modeling biodiversity with well-constructed ontologies

Although Figures 3 and 4 illustrate many possible biodiversity inputs and outputs that need tracking, they only scratch the surface of the use cases that can be modeled using carefully constructed ontologies in the biodiversity and ecology domains (Table S1). Biodiversity investigations often involve the collection of BCO:*material samples*, as in botanical or zoological collecting expeditions, species inventories or bio-blitzes, documentation of species at ecological observatories, ocean water sampling, and environmental sampling. In each of these use cases, material samples must be linked to data associated with the original collecting event as well as downstream derivatives such as duplicate specimens, DNA subsamples, photographs, or digital records.

Other use cases relevant to biodiversity studies will involve the collection of material samples, but not their preservation. A case in point is certain metagenomic studies, where biological specimens are effectively consumed or destroyed during the sampling processing. Metagenomic analyses present many other new challenges, given the large number of sequences that have no reference to taxonomic names. How can phylogenetic trees or operational units be easily combined across analyses [62]? How can the trees and operational units be reconciled with names and specimens? It becomes even more challenging when microbial communities exchange their genes between sampling/sequencing events, yielding new suites of sequences that differ from previous time points. Consistent use of standardized ontology terminology and stable identifiers can help overcome these challenges by providing a way to track samples and data over time.

Finally, many biodiversity data sets reference neither specimens nor genes, but instead provide only a list of taxa observed in an area, or even of taxa not detected (absence data). Absence data are theoretically critical for ecological niche modeling but come with their own set of challenges, both scientific (e.g., how to specify the relevant spatial and temporal baseline for an absence [63]) and ontological (e.g., how to capture negative assertions to the effect that entities of a given type do not exist [64]). Vegetation plot surveys, transects, and monitoring activities such as annual surveys of ecological observatories (Table S1) are classic examples of data sets that hold a wealth of relatively inaccessible biodiversity data in highly dispersed, non-standardized repositories. As the BCO grows to encompass ecological survey and inventory data, it will provide a key piece of the infrastructure needed to integrate survey data more effectively via shared, linked, and well-understood terms.

The use of ontology terms and globally unique identifiers, as part of a linked data framework, provides the means to answer key questions not only within complex multi-institutional projects, such as the Moorea Biocode Project and OSD, or across large-scale e-infrastructure initiatives such as the Genomic Observatories Network, but also within and among single-investigator led research projects or across citizen science initiatives. For example, a query such as “find all metagenomes collected from insects found in soil” requires data from many sources to be linked and freely available. Ontologies are essential to resolve queries such as this effectively, because data from different projects are often annotated with different levels of precision. For example, ENVO's *environmental material* hierarchy would allow this query to return results for samples collected in ENVO:*loam*, knowing that it is a subclass of ENVO:*soil*.

### Coordination with other ontologies and vocabularies

Curators of the BCO, ENVO, and PCO are committed to development following OBO Foundry principles. These include providing human readable textual definitions of terms, using consistent conventions for naming, formatting, versioning, and URI specification, and maintaining ontologies in light of scientific advances in the relevant domains. OBO Foundry principles are geared toward constructing a set of open access, interoperable, non-redundant ontologies built on shared content, collaboration, and documentation. The use of a shared upper ontology and common relations facilitates linking classes and instances between BCO, ENVO, PCO, and the suite of other ontologies being developed according to OBO Foundry principles. The BCO is able to re-use terms from OBO Foundry ontologies like GO [50], SO [52], OBI [54], or various anatomy ontologies to create an application ontology tailored to the needs of the biodiversity community (for example, Figures 3 and 4 show examples of integration of BCO and OBI). Developers also can take advantage of the methods and technologies developed in large-scale informatics projects that use OBO Foundry ontologies, such as the Neuroscience Information Framework (NIF) [14] and eagle-i [15].

To reduce redundancy, BCO, ENVO, and PCO each import a number of terms from other, independently developed ontologies. The BCO and PCO import the entirety of the BFO and the IAO's ontology-metadata ontology, plus CARO:*organism or virus or viroid* from the Common Anatomy Reference Ontology (CARO) [65]. BFO [48], [49] is an upper-level ontology that provides a formal, domain neutral specifications of basic types of entities such as BFO:*object*, BFO:*quality*, and BFO:*process*. IAO terms are used to provide annotation properties, as well as the term IAO:*information content entity* and its subclasses. This includes IAO:*data item*, which covers information generated as a result of an assay, such as a DNA sequence as found in Genbank. Descriptions of BCO:*material samples* from organismal parts required to supplement ENVO can be taken from taxon-specific anatomy ontologies as needed.

As development of the BCO progressed, it became clear that the notions of material sample, subsample, and measurement were already covered in the Ontology for Biomedical Investigations (OBI). However, given its much broader scope (OBI is an ontology that aims to enable the description of all biological and clinical investigations through a shared vocabulary [54]), OBI contains many unfamiliar term names and extraneous classes. A first step toward alignment among the BCO, OBI, DwC, and MIxS was the proposal by BCO developers to replace BCO:*material sample* in future versions with OBI:*specimen* and simultaneously propose its adoption by both DwC and MIxS. A formal proposal has been submitted to TDWG to add a new term called dwctype:MaterialSample (referencing OBI:*specimen*) to the DwC type vocabulary. A separate proposal, currently under review by the MIxS community, recommends a revision to the definition of the MIxS property source_mat_id that includes a reference to OBI:*specimen*. These proposals do not aim to replace the use of terms from DwC or MIxS; rather, adding “material sample” to those vocabularies allows information from them to filter up to BCO and OBI more effectively and provides a mechanisms for computationally accessing legacy biodiversity data sets annotated using DwC or ABCD (for collections data) or MIxS (for genomic and metagenomic data). Work is underway to convert legacy data sets annotated with DwC and MIxS to ontology-based data sets, and will be reported in a future publication, along with mappings of DwC and MIxS terms to BCO and other ontologies.

While the efforts described herein focus primarily on harmonization with vocabularies and ontologies from the life sciences, there are other communities actively developing knowledge representations for information collected by a broad range of earth science researchers. For example, the Open Geospatial Consortium offers a standard for describing environmental features, observations, and measurements in a formal XML schema (ISO/DIS 19156) that is being translated into RDF. Open Geospatial Consortium has also developed GeoSPARQL for querying geospatial data expressed in RDF. Development of the ESIP-based Semantic Web for Earth and Environmental Technology, or SWEET ontology is ongoing. The Scientific Observations Network, or SONet effort, is an NSF-funded INTEROP that is attempting to harmonize how observations and measurements are described in the context of ecological and environmental science investigations [66]. BCO curators have established communication with members from these communities as well, in order to achieve interoperability of semantic terminologies for natural science investigations in the broadest sense.

### Conclusions

The development of ontologies for biodiversity sciences aims to overcome several shortcomings of the current state of affairs: 1) a lack of clarity in the definitions of terms currently used for biodiversity data, 2) the inability to reason over complex data sets due to a lack of well-structured logical definitions, and 3) an inability to integrate museum collection data with other large biological data sets such as the GO database, environmental or metagenomic data, and survey-based data. However, these efforts can only realize their full potential when data are both digitized and shared. The development of ontologies must therefore go hand in hand with the ongoing digitization of biological collections and the development of online, sustainable data repositories that maintain stable, globally unique identifiers for data objects. Also critically important is the development of accessible tools to enable scientists to annotate their data accurately with terms drawn from ontologies and query their data using semantically enhanced techniques. These tools ideally will be integrated with the major data repositories supporting biodiversity investigations.

By providing a single unified structure for biodiversity knowledge – as opposed to *ad hoc* solutions that must be customized for each data set – the BCO and related ontologies permit potentially unlimited queries *across* data sets. This does not prohibit the construction of application-specific databases, but instead suggests that those databases should use ontology terms and URIs to make their data discoverable and interoperable; rather than replace existing vocabularies like DwC, the BCO supplements them. The success of the ontology-based, linked data approach that we propose depends on the adoption and review of BCO, PCO, and ENVO by the scientific community. We hope that current work will spur interest and feedback from scientists and bioinformaticians who see data integration, interoperability, and reuse as the solution to bringing the past 300 years of biological exploration of the planet into currency for science and society.

## Supporting Information

Table S1 Example use cases in biodiversity science that could be annotated using the BCO, ENVO, and/or PCO. Each of these use cases requires linking information (i.e. data or metadata) about material entities of interest to biologists as materials and data move through various processes and institutions. Examples are provided of the types of queries that can be facilitated through the use of ontologies, as well as links to example datasets.
(DOCX)
