# Supplementary material for: Semantics in Support of Biodiversity Knowledge Discovery: An Introduction to the Biological Collections Ontology and Related Ontologies
Source: PLoS One. 2014 Mar 3;9(3):e89606. doi: 10.1371/journal.pone.0089606 (PMC3940615; doi:10.1371/journal.pone.0089606)
Supplement: Table S1 — Example use cases in biodiversity science that could be annotated using the BCO, ENVO, and/or PCO. Each of these use cases requires linking information (i.e. data or metadata) about material entities of interest to biologists as materials and data move through various processes and institutions. Examples are provided of the types of queries that can be facilitated through the use of ontologies, as well as links to example datasets. (DOCX) [file pone.0089606.s001.docx]

**Table S1: Example use cases in biodiversity science that could be annotated using the BCO, ENVO, and/or PCO.** Each of these use cases requires linking information (i.e. data or metadata) about material entities of interest to biologists as materials and data move through various processes and institutions. Examples are provided of the types of queries that can be facilitated through the use of ontologies, as well as links to example datasets.

*Comprehensive species inventory of a park – Bio-blitz*

**Description:** A project aims to sample comprehensively all plants, animals, and fungi from a park. Sampling takes place over several days and across multiple habitats. Specimens of all animals are sent to one museum collection, while all plants and fungi are sent to a herbarium. Metadata are recorded about the habitat in which each specimen was found.

**Example queries:** List all plant species found in the meadow. List all invertebrates collected in the pond. Have any of the specimens from this project been subsampled for DNA sequencing?

**Example data sets:** [iNaturalist](http://www.inaturalist.org/); National Geographic [BioBlitz Projects](http://www.nationalgeographic.com/explorers/projects/bioblitz/)

*Botanical collecting expedition*

**Description:** A typical botanical collecting event results in a set of herbarium specimens that have numbers assigned by one or more collectors as well as IDs (barcodes) assigned by the herbarium. Duplicate specimens may be sent to alternate herbaria, with the same collector’s number, but possibly different herbarium IDs. Tissue samples from a subset of the individuals sampled may be collected and stored in silica for later DNA isolation, and alcohol preserved fruits or seeds from some individuals also may be submitted to the herbarium. Sometimes specimens are too large for a single herbarium sheet and require two or more barcodes that need to reference the same individual. During the collecting process, collectors may take images of live plants in their habitat and submit those to the herbarium database or an image repository. Herbarium specimens may later be digitized, and their images and associated data made available through the herbarium database. Specimen data may be submitted to GBIF. A similar workflow can be described for animal or fungal collecting expeditions.

**Example queries:** List the taxa associated with a specific collecting event. For a DNA sequence located through GenBank, list the date and time of collection and geographic coordinates. Locate all sheets in all herbaria that represent a single individual. Find the herbarium specimen associated with an image of a plant taken in the field.

**Example data sets:** Integrated Digitized Biocollections ([iDigBio](http://portal.idigbio.org/)) Portal for digitized specimens; any herbarium database, such as the [C.V. Starr Virtual Herbarium](http://sciweb.nybg.org/science2/VirtualHerbarium.asp) of the New York Botanical Garden; Global Biodiversity Information Portal ([GBIF](http://data.gbif.org/welcome.htm)) records

*Evidence of organisms in situ – citizen science*

**Description:** Traditionally, a museum specimen was considered the only valid evidence of the presence of an organism at a given time and location. However, the use of images and verbal reports to document biodiversity is becoming more common, not only by experts, but also as part of citizen science projects, where data are collected by people who may or may not be experts in the taxonomy of the organisms they are identifying. To support biodiversity research, reports of species occurrences must have metadata pertaining to the taxon, collecting event, and level of expertise of the person recording the data.

**Example queries:** List all known instances of the butterfly genus *Danaus* from North America during the year 2012, sorted by evidence type (museum collection, image, verbal report). List all organisms known to occur in Pima County, Arizona.

**Example data sets:** Monarch butterfly monitoring projects such as the [Monarch Larval Monitoring Program](http://www.mlmp.org/), North American Butterfly Association ([NABA](http://www.naba.org/)) butterfly counts, and the [Journey North](http://www.learner.org/jnorth/monarch/index.html) program for K-12 students; [Morphbank](http://www.morphbank.net/About/AboutMb/) image repository; [iNaturalist](http://www.inaturalist.org/); [eBird](http://ebird.org/content/ebird/about/) bird observation data; National Audubon Society’s [Christmas Bird Count](http://birds.audubon.org/christmas-bird-count)

*Ecological Observatories*

**Description:** A number of field stations have become “ecological observatories”, that is, locations on the earth where long-term data on species composition and abundance are recorded (i.e. monitored), often along with data on community dynamics or ecosystem function. DNA barcode, genomic, or metagenomic data may be collected at the same locations. Some ecological observatories are grouped together into networks to share data collection protocols, informatics infrastructure, or broad-scale research questions.

**Example queries:** List all observatories that collect data on plant-pollinator interactions. Find all known locations of *Quercus* where CO_2_ flux data have been recorded for a specific period. Find metagenomic sequence data associated with fresh water aquatic field stations that also collect data on water quality.

**Example data sets:** Long Term Ecological Research ([LTER](https://metacat.lternet.edu/das/lter/browse.jsp)) Network data portal; Smithsonian Tropical Research Institute ([STRI](http://biogeodb.stri.si.edu/bioinformatics/en/)) databases; biodiversity data from National Ecological Observatory Network ([NEON](http://www.neoninc.org/science/data)); [Forestplot.net](http://www.forestplots.net/en/plot-information) forest monitoring data; [Hubbard Brook Forest](http://www.hubbardbrook.org/data/dataset_search.php) Ecosystem Study

*Ocean water sampling*

**Description:** Marine research stations and individual expeditions collect ocean water at regular locations at regular intervals, following standardized protocols. A known volume of water is collected from a specific depth, and aliquots taken from the primary sample. Different aliquots are filtered at different pore sizes, to collect different classes of organisms, which are then counted and identified, often only to the genus or family level. Metagenomic sequencing may be performed on a subsample of the water, resulting in a list of microbial names based on sequences matched to a database (see *Environmental sequencing*, below). In addition to biotic sampling, aliquots of the water may be analyzed for chemical parameters such as nutrient or dissolved oxygen content.

**Example queries:** Are there differences in microbial community composition of artic sea water at 5 M versus 10 M depth? Compare the planktonic composition of ocean water samples collected in areas of high versus low iron content. For a specific metagenomic data set, find the date, time, and vessel associated with the original water sample and list any other data sets associated with the same sample.

**Example data sets:** Micro B3 [Ocean Sampling Day](http://www.microb3.eu/osd) data; plankton datasets from the [Arctic Ocean Diversity](http://www.arcodiv.org/Database/Plankton_datasets.html) project; Ocean Biogeographic Information System ([OBIS](http://www.iobis.org/)) database

*Environmental sampling*

**Description:** Environmental sampling involves collecting samples of environmental materials such as water or soil, extracting the microbial community from the material, and bulk sequencing of genomic DNA. At the time of collection, data on the location and environmental parameters of the sample are recorded. The original material sample is often completely destroyed during later processing steps. Sequences are queried against databases of known sequences to produce a list of taxa present in the sample. If the environmental sample is stored before sequencing, the microbial community may change over time, with the result that sequences taken from the same sample at different time points may represent different communities. The output sequences can also vary depending on sampling, extraction, and sequencing protocols.

**Example queries:** Find all metagenomic sequences that were produced by a specific sequencing protocol. Compare the microbial community composition of environmental soil samples from arid versus mesic environments. List all locations where a specific microbial taxon is known to occur.

**Example data sets:** Micro B3 [Ocean Sampling Day](http://www.microb3.eu/osd) data; Genomes Online Database ([GOLD](http://www.genomesonline.org/cgi-bin/GOLD/index.cgi)); Community Cyberinfrastructure for Advanced Microbial Ecology Research and Analysis ([CAMERA](http://camera.calit2.net/))

*Organismal metagenomic sampling*

**Description:** Organisms are sampled for metagenomic sequencing of the microbiome of their gut, nasal passage, leaf surface, or other location. Sample collection and sequencing follow specific protocols. For wild animals, there are protocols associated with the capture or collection of an animal, and data are collected on the environmental conditions in which the animal was found. For captive animals or humans, laboratory conditions or health status may be recorded. A sample of tissue may be collected at the same time for DNA isolation and gene sequencing, and part of or the entire organism may be submitted to a museum for preservation.

**Example queries:** Find all metagenomic data sets associated with insects collected in tropical rain forests. Compare the differences in community composition of the gut flora of patients with Celiac disease versus healthy patients. Locate the museum specimens associated with a set of organismal metagenomic samples.

**Example data sets:** Genomes Online Database ([GOLD](http://www.genomesonline.org/cgi-bin/GOLD/index.cgi)); Community Cyberinfrastructure for Advanced Microbial Ecology Research and Analysis ([CAMERA](http://camera.calit2.net/)); Human Microbiome Project ([HMP](http://www.hmpdacc.org/resources/data_browser.php))

*Vegetation plot survey*

**Description:** Plot-based vegetation surveys are used to quantify plant species diversity in an area (e.g., a forest or a biological reserve). One or more plots of fixed size are chosen, either randomly or as examples of representative vegetation, and surveyed according to predetermined protocols. Plot size will vary, depending on the type of vegetation to be surveyed (i.e. larger plots for trees and smaller plots for herbs). Plots may be divided into sub-plots and sub-sub-plots, and different types of vegetation may be counted at different levels. Often, the exact number of trees above a certain size is counted, while diversity of the herbaceous layer is measured as percent cover of different species. In addition to presence, individual trees may be measured for their size (e.g., diameter at breast height or DBH). Additional information such as the reproductive state or vegetative phenology of individuals or species may also be recorded. The presence of rare species may be recorded even if they fall outside the plot area. In some cases, the absence of certain species is also recorded. From the survey data, different measures of species richness or diversity may be calculated

**Example queries:** List all locations that have high abundance of *Solidago*. Find all locations where a particular species is known to be absent. List all biosphere reserves that have accurate lists of tree species present in the reserve.

**Example data sets:** Global Index of Vegetation Plot Databases ([GIVD](http://www.botanik.uni-greifswald.de/world_index_vegdb0.html)); U.S. National Park Service [Vegetation Inventory Program](http://science.nature.nps.gov/im/inventory/veg/plots.cfm); [Forestplot.net](http://www.forestplots.net/en/plot-information) forest monitoring data

*Butterfly or bird transect survey*

**Description:** A transect is a predetermined linear path (that may be straight or sinuous) through one or more habitats. Researchers walk a transect and record all sightings of selected taxa (e.g., all birds, all butterflies) within a set distance (e.g., 5 or 10 meters) of the transect. In addition to recording every individual sited, data are also recorded on parameters such as survey effort (how many people for how many hours), habitat(s), weather conditions, time of day, or season. Sometimes particular taxa may be excluded from the survey (e.g., all birds except raptors). Often, the same transect is surveyed repeatedly during the course of a year or season, to create a list of known species at a given location and allow researchers to infer the absence of species not recorded.

**Example queries:** List all know locations of *Cardinalis cardinalis* associated with a transect survey, along with any species known to co-occur with it. Do forest biomes have higher or lower butterfly diversity than meadow biomes? Find any ecological research stations that have data on both vegetation plots and butterfly transect surveys.

**Example data sets:** Rocky Mountain Bird Observatory ([RMBO](http://rmbo.org/v3/avian/DataCollection.aspx)) Avian Data Center; United Kingdom [Butterfly Monitoring Scheme](http://www.ukbms.org/Methods.aspx); U.S. Geological Survey [Northern Prairie Wildlife Research Center](http://www.npwrc.usgs.gov/resource/insects/butsurv/intro.htm) butterfly monitoring system
